# Clinical and molecular characterization of *KCNT1*-related severe early-onset epilepsy

**DOI:** 10.1212/WNL.0000000000004762

**Published:** 2018-01-02

**Authors:** Amy McTague, Umesh Nair, Sony Malhotra, Esther Meyer, Natalie Trump, Elena V. Gazina, Apostolos Papandreou, Adeline Ngoh, Sally Ackermann, Gautam Ambegaonkar, Richard Appleton, Archana Desurkar, Christin Eltze, Rachel Kneen, Ajith V. Kumar, Karine Lascelles, Tara Montgomery, Venkateswaran Ramesh, Rajib Samanta, Richard H. Scott, Jeen Tan, William Whitehouse, Annapurna Poduri, Ingrid E. Scheffer, W.K. “Kling” Chong, J. Helen Cross, Maya Topf, Steven Petrou, Manju A. Kurian

**Affiliations:** From Molecular Neurosciences (A.M., E.M., A., A.N., M.A.K.), Developmental Neurosciences, UCL Great Ormond Street Institute of Child Health; Department of Neurology (A.M., A., A.N., C.E., J.H.C., M.A.K.) and Neuroradiology (W.K.C.), Great Ormond Street Hospital for Children, London, UK; Florey Institute of Neuroscience and Mental Health (U.N., E.V.G., I.E.S., S.P.), Melbourne, Australia; Department of Biological Sciences (S.M., M.T.), Institute of Structural and Molecular Biology, Birkbeck College, University of London; Regional Molecular Genetics Laboratory (N.T., R.H.S.), North East Thames Regional Genetics Service, and Department of Clinical Genetics (A.V.K., R.H.S.), Great Ormond Street Hospital, London, UK; Department of Paediatric Neurology (S.A.), Red Cross War Memorial Children's Hospital, Cape Town, South Africa; Department of Paediatric Neurology (G.A.), Addenbrooke's Hospital, Cambridge; Roald Dahl EEG Unit (R.A.), Department of Neurology, and Department of Neurology (R.K.), Alder Hey Children's Hospital, Liverpool; Department of Paediatric Neurology (A.D.), Sheffield Children's Hospital; Clinical Neurosciences (C.E., J.H.C.), Developmental Neurosciences, UCL Great Ormond Street Institute of Child Health, London; Institute of Infection and Global Health (R.K.), University of Liverpool; Department of Paediatric Neurology (K.L.), Evelina Children's Hospital, Guys and St. Thomas' NHS Foundation Trust, London; Department of Clinical Genetics (T.M.), Northern Genetics Service; Department of Pediatric Neurology (V.R.), Great North Children's Hospital, Newcastle Upon Tyne; Department of Paediatric Neurology (R.S.), University Hospital Leicester Children's Hospital; Department of Paediatric Neurology (J.T.), Royal Manchester Children's Hospital; Department of Paediatric Neurology (W.W.), Nottingham University Hospitals NHS Trust, UK; Epilepsy Genetics Program (A. Poduri), Department of Neurology, Boston Children's Hospital; Department of Neurology (A. Poduri), Harvard Medical School, Boston, MA; University of Melbourne (I.E.S.), Austin Health and Royal Children's Hospital, Australia; and Department of Medicine (S.P.), Royal Melbourne Hospital, University of Melbourne, Australia. Dr. Malhotra is currently at the Department of Biochemistry, University of Cambridge, UK.

## Abstract

**Objective:**

To characterize the phenotypic spectrum, molecular genetic findings, and functional consequences of pathogenic variants in early-onset *KCNT1* epilepsy.

**Methods:**

We identified a cohort of 31 patients with epilepsy of infancy with migrating focal seizures (EIMFS) and screened for variants in *KCNT1* using direct Sanger sequencing, a multiple-gene next-generation sequencing panel, and whole-exome sequencing. Additional patients with non-EIMFS early-onset epilepsy in whom we identified *KCNT1* variants on local diagnostic multiple gene panel testing were also included. When possible, we performed homology modeling to predict the putative effects of variants on protein structure and function. We undertook electrophysiologic assessment of mutant KCNT1 channels in a *xenopus* oocyte model system.

**Results:**

We identified pathogenic variants in *KCNT1* in 12 patients, 4 of which are novel. Most variants occurred de novo. Ten patients had a clinical diagnosis of EIMFS, and the other 2 presented with early-onset severe nocturnal frontal lobe seizures. Three patients had a trial of quinidine with good clinical response in 1 patient. Computational modeling analysis implicates abnormal pore function (F346L) and impaired tetramer formation (F502V) as putative disease mechanisms. All evaluated *KCNT1* variants resulted in marked gain of function with significantly increased channel amplitude and variable blockade by quinidine.

**Conclusions:**

Gain-of-function *KCNT1* pathogenic variants cause a spectrum of severe focal epilepsies with onset in early infancy. Currently, genotype-phenotype correlations are unclear, although clinical outcome is poor for the majority of cases. Further elucidation of disease mechanisms may facilitate the development of targeted treatments, much needed for this pharmacoresistant genetic epilepsy.

Autosomal dominant pathogenic variants in *KCNT1*, encoding the sodium-activated potassium channel, are identified in a wide spectrum of epileptic disorders with variable age at onset and cognitive outcome. These include severe early-onset epileptic encephalopathies such as Ohtahara and West syndromes^1,2^ and epilepsy of infancy with migrating focal seizures (EIMFS),^3–14^ as well as autosomal dominant and sporadic severe nocturnal frontal lobe epilepsies (ADNFLE and NFLE),^10,15,16^ but the genotype-phenotype relationship appears to be unclear. We undertook detailed clinical, molecular genetic, and functional characterization of a cohort of patients with *KCNT1*-related epilepsy.

## Methods

### Patient recruitment

We recruited patients with EIMFS (n = 31) to a research study investigating the genetic basis of early-onset epileptic encephalopathy (EOEE) between 2011 and 2016, following an earlier national surveillance study.^4^ Inclusion criteria were epilepsy with onset at <2 years and unknown etiology. Diagnostic criteria for EIMFS were as described in the previous study.^4^ Patients were recruited at Great Ormond Street Hospital, London, UK, and by referral from other centers in the United Kingdom and internationally. Two patients who had routine local diagnostic multiple gene panel testing revealing *KCNT1* variants were also included.

### Standard protocol approvals, registrations, and patient consents

We obtained written informed consent from families in whom research genetic investigations were undertaken. The study was approved by the National Research Ethics Service (London-Bloomsbury, Research Ethics Committee reference 13/LO/0168, Integrated Research Application System project identifier 95005). We collected anonymized data from patients tested on the diagnostic next-generation sequencing panel (n = 3) as part of an approved case note review project (Great Ormond Street Hospital Research and Development Department, 16NM11).

### Genetic testing

We used a variety of different methods (table e-1, http://links.lww.com/WNL/A6), including direct Sanger sequencing, multiple gene panel testing with the TruSeq Custom Amplicon panel and SureSelect panel, exome sequencing (e-Methods, http://links.lww.com/WNL/A8; tables e-1 and e-2, http://links.lww.com/WNL/A6), and diagnostic chromosomal microarray.

### Homology modeling

HMMscan^17^ against Pfam (database of sequence-based domain families)^18^ identified 2 domains in the sequence of human KCNT1 (isoform 1): ion channel (PF07885, at position 278–346) and calcium-activated BK potassium channel alpha-subunit family (PF03493, at position 495–598) (e-Methods).

### Electrophysiologic assessment of mutant KCNT1 in *xenopus* oocyte model

We introduced variants into a wild-type (WT) human *KCNT1* expression construct^19^ using QuikChange Lightning Site-Directed Mutagenesis Kit (Agilent Technologies, Santa Clara, CA). cDNAs were transcribed in vitro (mMessage mMachine; Ambion, Austin, TX). Oocytes were prepared, and 2-electrode voltage clamp recording was performed after 14 to 24 hours of expression. We also recorded currents before and after the application of Quinidine (e-Methods).

## Results

### Clinical and molecular genetic features of *KCNT1* mutation–positive patients

#### Clinical presentation

We identified pathogenic variants in *KCNT1* in 12 patients, 5 through direct Sanger sequencing, 2 from whole-exome sequencing, and 5 from the Great Ormond Street Hospital diagnostic panel (5 of 800 tested patients with EOEE/developmental delay).

**Table 1:**
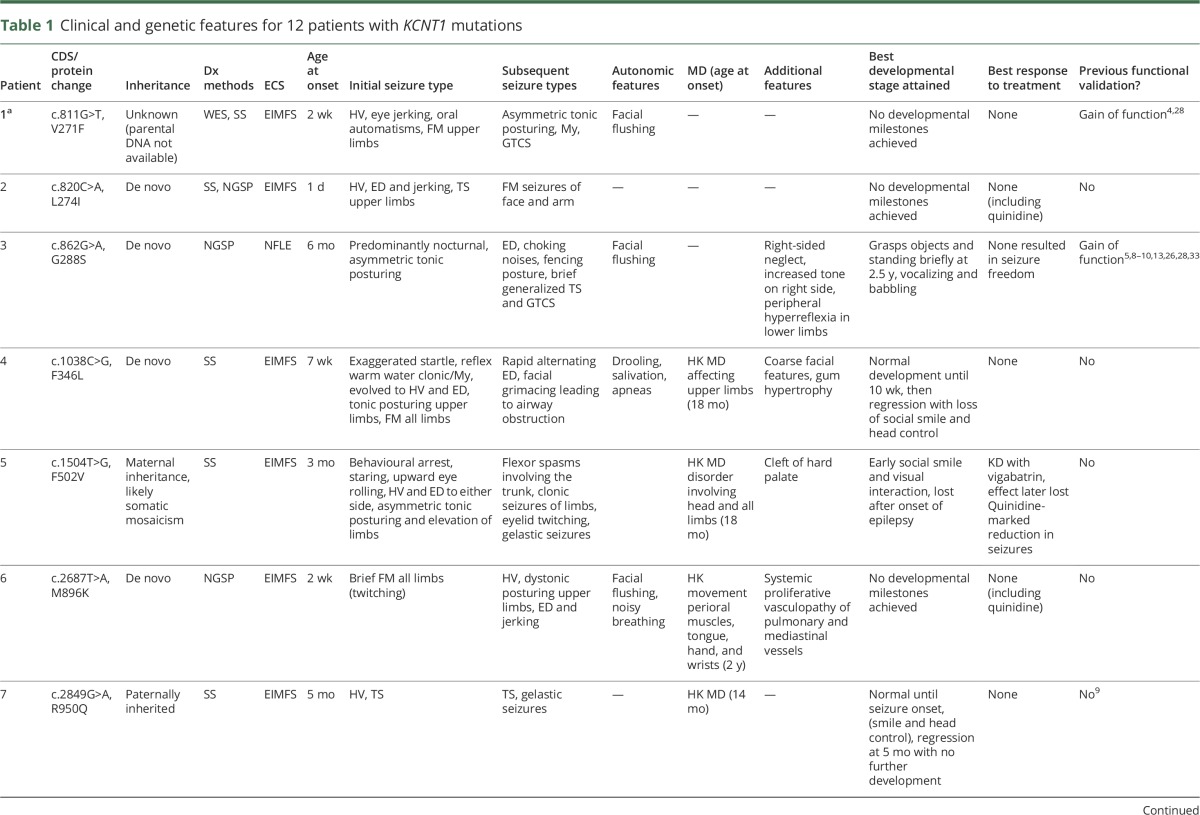

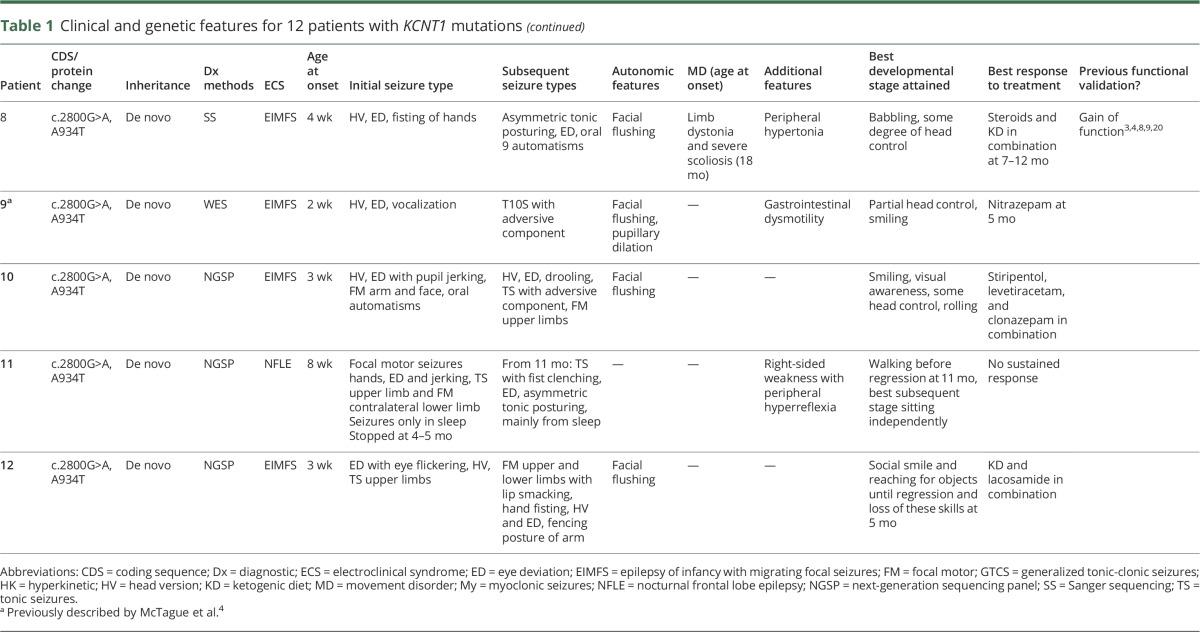
Clinical and genetic features for 12 patients with *KCNT1* mutations

Clinical features are summarized in table 1. Median age at seizure onset was 3.5 weeks (range 1 day–6 months). Most patients developed seizures consistent with EIMFS. Two patients (patients 3 and 11) presented with severe, early-onset NFLE, characterized by asymmetric tonic posturing and later fencing posture. We noted similar frontal seizure semiology in patients with EIMFS (e.g., patient 12). All patients developed axial hypotonia, and upper motor neuron signs emerged in 3 patients. Four patients had a choreiform movement disorder (onset 14–24 months); 1 patient developed generalized dystonia at 18 months. Onset of hyperkinesia was not related to medication (including vigabatrin) nor triggered by intercurrent illness. Initial age at presentation, disease course, response to medication, brain MRI, and EEG findings were similar for both *KCNT1* pathogenic variant–positive and –negative patients from the cohort. However, 5 of 12 pathogenic variant–positive patients with EIFMS presented with a severe movement disorder compared to 2 of 19 *KCNT1*-negative cases. Most had extensive uninformative laboratory metabolic and genetic investigations. Abnormal muscle respiratory chain enzyme activity for complex I and/or II was detected in patients 4 and 8 of uncertain significance (table e-3, http://links.lww.com/WNL/A6). For patient 4, the muscle biopsy was taken during an intercurrent illness and repeated after clinical recovery, revealing a more borderline result. In patient 8, borderline abnormalities in complex I and II ratios were found. Neither patient had other systemic, biochemical, or radiologic features of mitochondrial disease or concurrent sodium valproate treatment.

In general, neurodevelopmental outcome was markedly impaired in all patients with EIMFS. All patients had a trial of at least 5 different medications. Response to treatment was, in general, poor (table 1). Three of 8 patients who received the ketogenic diet in combination with other antiepileptic drugs responded with ≈75% seizure reduction. Three patients were treated with quinidine. Patient 2 received 40 mg/kg/d without adverse events but with no effect on seizure burden. Patient 5 was treated with quinidine at 40 mg/kg/d, leading to a marked reduction in seizure frequency. Patient 6 showed some initial transient reduction in seizure frequency at 30 mg/kg/d. For this patient, the unexpected development of a severe proliferative pulmonary and mediastinal vasculopathy resulted in life-threatening pulmonary hemorrhage. Investigations failed to identify an underlying vasculitis, and quinidine was subsequently withdrawn. The patient later died despite initial successful pulmonary embolization.

#### EEG features

All patients with an EIMFS phenotype had a “migrating” ictal focus with discrete ictal involvement of differing cortical areas within the same EEG (table e-4, http://links.lww.com/WNL/A6). Although not always evident at initial presentation, it developed by 7 months of age in most patients. Periods of EEG suppression or burst suppression were noted in 8 of 12 patients; 6 of these patients had seizure onset in the first 4 weeks of life. Further atypical EEG features included a generalized electrodecremental response in 5 patients and hypsarrhythmia in 1 patient.

#### Radiologic features

Neuroimaging was available for review in 11 of 12 patients. The majority developed predominantly frontal cerebral atrophy by 3 years of age (figures e-1A and e-1B, http://links.lww.com/WNL/A7; table e-5, http://links.lww.com/WNL/A6). Cerebellar atrophy was also evident in 4 patients (figure e-1C). We noted an open operculum in the first 6 months of life in patient 4 (figure e-1B). Delayed myelination was evident in 9 of 11 patients who had imaging after 3 months of age. In some patients, early brain imaging was normal. Magnetic resonance spectroscopy was abnormal with a relatively reduced N-acetylcholine peak in 3 of 4 patients.

#### Molecular genetic findings

We identified 12 patients with pathogenic variants in *KCNT1* (table 1 and table e-6, http://links.lww.com/WNL/A6); 8 have been previously reported and 4 are unpublished.^4,5,9,10,16^ Eight of 12 patients had C-terminus variants, of whom 5 had the commonly reported variant A934T. We have identified 4 (including 2 unpublished) pathogenic variants causing EIMFS, namely V271F, L274I, G288S, and F346L located in or between transmembranes 5 and 6. All are missense variants that are predicted to be pathogenic (table e-6), affecting highly conserved amino acid residues (figure e-2, http://links.lww.com/WNL/A7), and are not reported in 1000 Genomes, the ExAC database, or the Exome Variant Server.^20–24^ For 9 of 12 cases, variants occurred de novo. Parental DNA was not available for patient 1. In patient 5, we found the same *KCNT1* variant in an asymptomatic mother and her affected child. We noted a lower heterozygous peak on Sanger sequencing of both salivary and blood-derived maternal genomic DNA (figure e-3), which may reflect somatic mosaicism. In patient 7, the variant was inherited from the unaffected father with no difference in peak size on Sanger sequencing (figure e-4). The recurrent A934T variant was identified in 5 patients, 4 with an EIMFS presentation and 1 (patient 11) with an NFLE phenotype.

### Protein homology modeling of mutant KCNT1

**Figure 1:**
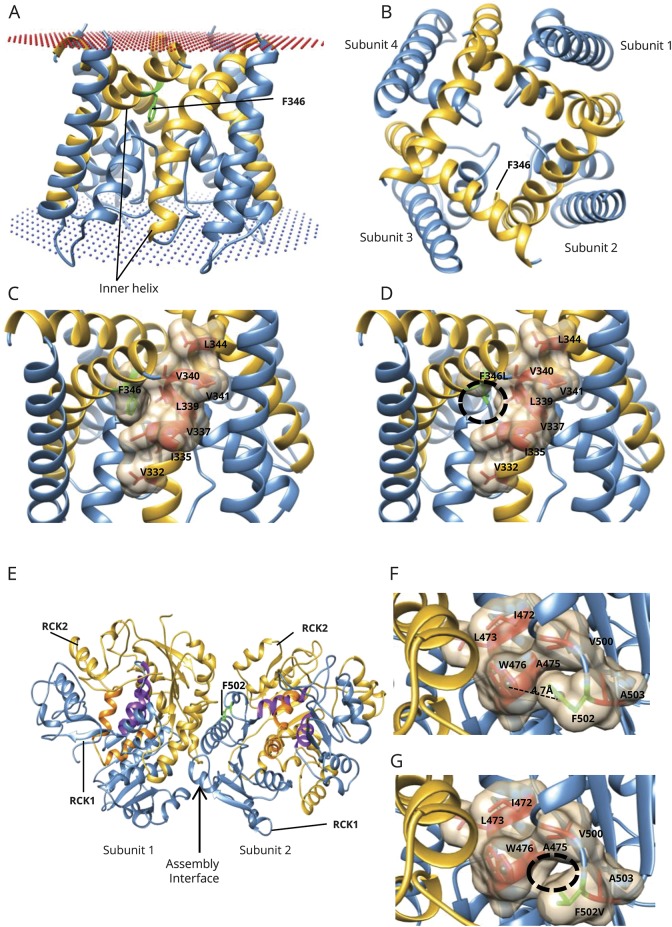
Modeling the ion channel and gating apparatus of *KCNT1* (A) Side view of the homology model of the KCNT1 ion channel (residues 278–346) as a tetramer. F346 is present on the edge of the inner helix (in gold) and interacts with the inner helix of the adjacent subunit in the tetrameric arrangement. Membrane position is shown in spheres. (B) Top view of the tetramer arrangement of the ion channel and location of F346 on the inner helix. (C) F346 is part of the hydrophobic cavity (shown as surface), which mediates interactions between the inner membrane helices of the 2 subunits. F346 is shown in green; the surrounding hydrophobic residues are shown in red. (D) On mutation to leucine (F346L, in green), the hydrophobic interactions between the 2 subunits are likely to be reduced (black circle) because the side chain of leucine is much shorter than phenylalanine. (E) Model of a dimer of the gating ring (residues 373–1,044; residues 1,045–1,174 could not be modeled), which is a tetramer (dimer of the modeled dimer). Each subunit possesses 2 RCK domains: RCK1 (in blue) and RCK2 (in gold). F502 (in green) is present in the RCK1 domain, near the intersubunit interface (assembly interface). The RCK1-RCK2 intrasubunit interface is purple (residues from RCK1) and orange (residues from RCK2). The dimer interfaces formed by both RCK-1 and RCK-2 are indicated by an arrow. (F) F502 (green) and its neighboring hydrophobic residues (red), including W476, with which it could potentially form a pi-pi interaction. Distance between the centroid (spheres) of the 2 rings (F502 and W476) is 4.7 Å, and the angle between the ring planes is 27.3°. (G) F502V could abolish the formation of the potential pi-pi interaction with W476 and is likely to reduce the hydrophobic interactions (black circle) because the side chain of valine is smaller than that of phenylalanine.

Homology modeling was performed for 2 novel mutations: F346L, located in the ion channel domain (residues 270–353), and F502V, located in the gating region (residues 373–1174, although residues 1,045–1,174 could not be modeled). F346 is located on the inner helix of the transmembrane pore (figure 1, A and B). It is part of the hydrophobic cavity, which mediates interactions between the inner-membrane helices of 2 adjacent subunits (figure 1C) and is thus responsible for maintaining the stability of the open conformation. In the modeled closed-state conformation, the helix containing F346 and the inner helix from the other protomer undergo conformational changes (figure e-5, http://links.lww.com/WNL/A7). Therefore, mutation to leucine (F346L) is likely to destabilize the open state by perturbing the hydrophobic interactions because the side chain of leucine is smaller (figure 1D), affecting the equilibrium between the closed and open states. In addition, the packing arrangement in the K+ channels involving the pore and the inner helix is known to be critical for the stability of the tetrameric assembly, ion conduction function, and cation selectivity. Thus, F346L might be detrimental to these functions.^25^

Within each protomer of the KCNT1 gating region, there are 2 tandem RCK domains (RCK1 and RCK2) that serve as regulators of potassium conductance (figure 1E). These form flexible intrasubunit and intersubunit (figure 1E) interfaces that facilitate functional tetramer formation.^26^ F502 is located in RCK1 and predicted to form a pi-pi interaction with W476 from αD (figure 1F). F502 is also surrounded by a number of hydrophobic residues (I472, L473, A475, V500, and A503), which may play a role in stabilizing the gating ring (figure 1F). The amino acid substitution F502V is predicted to result in destabilization of these hydrophobic interactions, given the smaller valine side chain (figure 1G), and abolition of potential pi-stacking with resultant disruption of the stable assembly interface.

### Electrophysiologic assessment of mutant KCNT1

**Figure 2:**
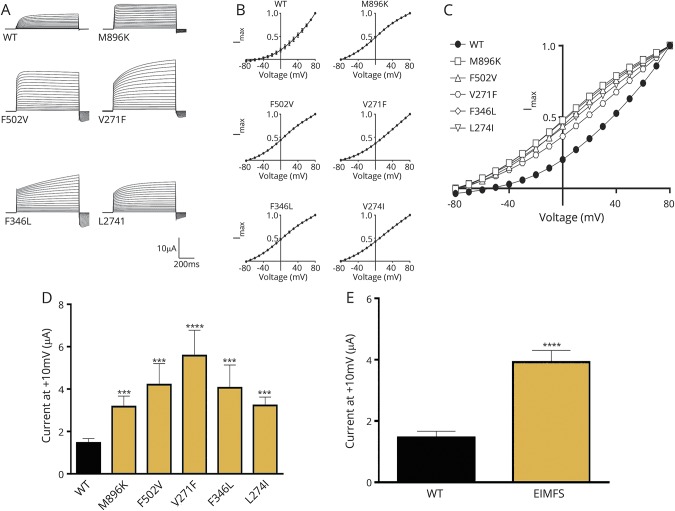
Functional investigation of *KCNT1* mutations in a *xenopus* oocyte model (A) Representative current traces obtained from oocytes expressing WT and EIMFS mutants (M896K, F502V, V271F, F346L, and L274I). Oocytes were held at −90 mV and stepped from −80 to 80 mV for 600 milliseconds every 5 seconds. Scale bars apply to all traces. (B) Current-voltage relationships for WT (n = 32), M896K (n = 15), F502V (n = 13), V271F (n = 9), F346L (n = 11), and L274I (n = 12). Currents were averaged and then normalized to the value at a test potential of 80 mV (*I*_max_). (C) Comparison of current-voltage relationships between WT (solid circles, n = 32) and EIMFS mutations (M896K [squares, n = 15], F502V [triangles, n = 13], V271F [hexagons, n = 9], F346L [diamonds, n = 11], and L274I [inverted triangles, n = 12]). Currents were averaged and then normalized to the value at a test potential of 80 mV (Imax). (D) Average peak currents at 10 mV for WT (n = 44), M896K (n = 19), F502V (n = 16), V271F (n = 10), F346L (n = 11), and L274I (n = 12) channels. Peak currents for each mutant channel at 10 mV were compared to the peak currents for the WT channel at 10 mV. ***p < 0.001, ****p < 0.0001. (E) Comparison of pooled WT (n = 44) and EIMFS (n = 68) currents at 10 mV. ****p < 0.0001. EIMFS = epilepsy of infancy with migrating focal seizures; WT = wild-type.

We evaluated the 4 previously unpublished variants and V271F, which we previously described^4^ and was recently studied in a *xenopus* oocyte system.^27^ All mutations resulted in an increased current magnitude compared to WT (figure 2A). We noted that for variants V271F and F346L, the rate of activation was slowed at higher voltages compared to WT, and in others (M896K, F502V and L274I), the activation rates were generally faster than WT (figure 2A). Investigation of the current-voltage relationship showed that mutant channels were very weakly voltage dependent, and in some cases, voltage dependence of steady-state activation was essentially absent (figure 2, B and C) with only a residual Goldman-Hodgkin-Katz rectification. Assessment of average peak currents at 10 mV revealed a significant difference between both individual mutant channels and summated data compared to WT (figure 2, D and E).

### Effect of 300 μmol/L quinidine on mutant KCNT1

**Figure 3:**
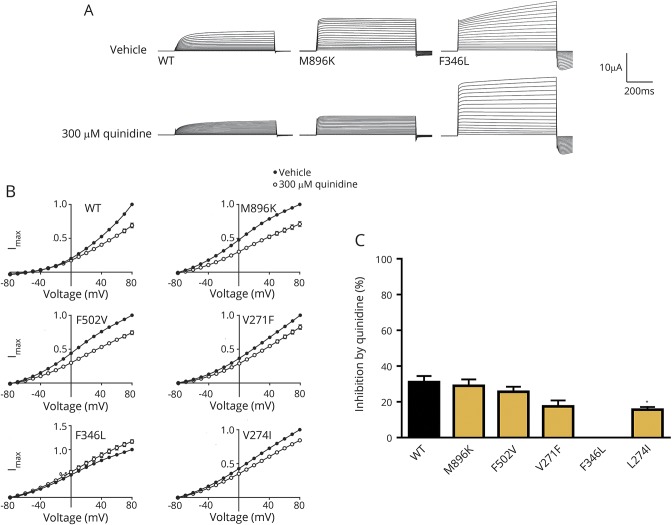
Effect of quinidine on *xenopus* oocytes expressing h*KCNT1* channels (A) Representative current traces obtained from oocytes expressing WT and EIMFS mutants (M896K and F346L) with application of vehicle (ND96) and 300 μmol/L quinidine. Oocytes were held at −90 mV and stepped from −80 to 80 mV for 600 milliseconds every 5 seconds. Scale bars apply to all traces. (B) Current-voltage relationships for WT (n = 32), M896K (n = 15), F502V (n = 13), V271F (n = 9), F346L (n = 11), and L274I (n = 12) h*KCNT1* channels in the presence of vehicle (ND96) and 300 μmol/L quinidine. Currents were averaged and then normalized to the value at a test potential of 80 mV (*I*_max_). (C) Average percent inhibition at 80 mV of WT (n = 31) and EIMFS (M896K, n = 15; F502V, n = 13; V271F, n = 9; F346L, n = 11; and; L274I, n = 12) h*KCNT1* channels by quinidine (300 μmol/L) depicting the variable degree of block by 300 μmol/L quinidine (1-way analysis of variance followed by Bonferroni post hoc analysis). *p < 0.1. EIMFS = epilepsy of infancy with migrating focal seizures; WT = wild-type.

Quinidine 300 µmol/L had variable current-blocking effects in different mutant channels. For F346L, peak current was completely insensitive to quinidine, although it had some effect on activation kinetics (figure 3A). The differential sensitivity of KCNT1 mutants to quinidine was clearly shown in the current-voltage relationship (figure 3B) and percentage of inhibition at maximum current, 80 mV (figure 3C). There is some correlation between the in vitro studies and clinical response in patient 5 (figure 3). M896K had the most marked in vitro blockade by quinidine, and patient 6 showed some initial clinical response. F346L showed no quinidine response at all, and the patient harboring this mutation was not treated with quinidine.

## Discussion

We report a cohort of patients with early-onset epilepsy associated with pathogenic variants in *KCNT1*, which encodes the sodium-activated potassium channel KCa4.1 (sequence like a calcium-dependent potassium channel [SLACK], Slo2.2). KCNT1 is widely expressed throughout the brain, as well as in the dorsal root ganglia, kidney, and heart, and is responsible for slow hyperpolarization after bursts of action potentials.^28,29^ KCNT1 also has direct interactions with Fragile X-related protein.^29^ Compared with other potassium channels, KCNT1 is involved in a highly extensive protein network, suggesting a putative role in cognitive developmental processes.^3,8,28,30^

To date, *KCNT1* variants have been reported in a wide range of epilepsies (table e-7, http://links.lww.com/WNL/A6).^1–16,31,32^ We identified patients with the same variant associated with varying electroclinical phenotypes (table 1). Phenotypic variability has been reported within single families in which different individuals may present with either ADNFLE or EIMFS.^10^ Such intrafamilial variation in phenotype is also described in *SCN1A* kindreds; Dravet syndrome, febrile seizures, and a variety of other generalized epilepsies may be reported in the same family.^33^ Furthermore, while the majority of variants in our cohort occurred de novo, 2 patients inherited variants from an unaffected parent. The mechanisms underlying phenotypic variability and true/apparent nonpenetrance are unclear but may be related to somatic mosaicism, variant type, other genetic/epigenetic factors, or differential expression of alternative *KCNT1* transcripts.^9,10,29,34,35^

The majority of patients with pathogenic *KCNT1* variants in our cohort had electroclinical EIFMS, although this is likely to reflect ascertainment bias. Indeed, 2 of the 5 *KCNT1*-positive patients identified by the diagnostic panel from a larger cohort of 800 patients with EOEE/developmental delay had an NFLE-like presentation. Although movement disorders are increasingly reported in other severe early-onset genetic epilepsies, they appear to be rare in *KCNT1* epilepsy.^36^ We describe several atypical EEG features. Generalized electrodecrement and hypsarrhythmia, more classically associated with infantile spasms, have been previously described in EIMFS.^2,4,9,10,31^ EEG suppression, classically seen in Ohtahara syndrome,^37^ has been only rarely described in EIMFS.^4,9^ Extensive diagnostic investigations undertaken in patients with *KCNT1* mutations were unyielding other than abnormal respiratory chain enzyme analysis of muscle tissue in 2 patients. The relevance of these findings is not clear, but secondary mitochondrial effects may be evident in *KCNT1* epilepsy, as often reported in other severe drug-resistant epilepsies.^38^ Other genetic and environmental influences on mitochondrial function may also play a role.

**Figure 4:**
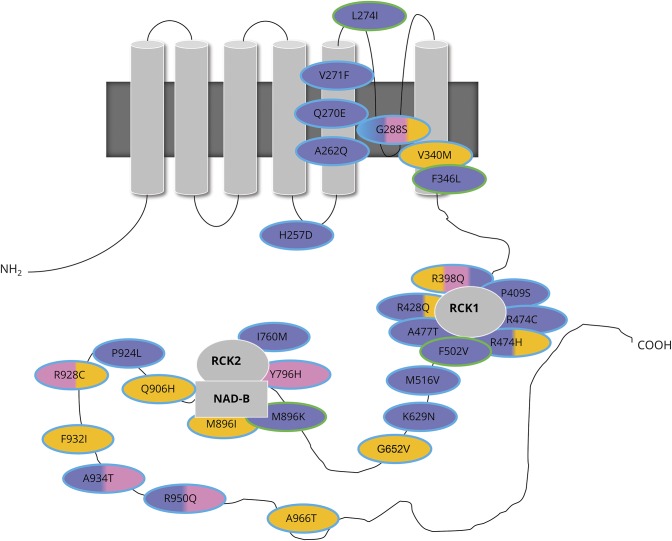
Schematic diagram of the location of mutations in *KCNT1* in this and previously published studies *KCNT1* encodes sequence like a calcium-dependent potassium channel (SLACK), which forms tetramers (top left) or heteromers with KCNT2 or sequence like an intermediate conductance K channel (SLICK). The structure comprises 6 transmembrane domains with a pore-forming region, regulator of potassium conductance (RCK), and nicotinamide adenine dinucleotide–binding (NAD-B) domains. EIMFS phenotypes are shaded in purple, ADNFLE or NFLE in pink, others (Ohtahara syndrome, leukoencephalopathy, focal epilepsy, EOEE, West syndrome, unaffected) in orange. Mutations giving rise to >1 phenotype are shaded with a combination of the corresponding colors. Novel mutations identified in this study are outlined in green, those identified in previous studies in turquoise. ADNFLE = autosomal dominant nocturnal frontal lobe epilepsy; EIMFS = epilepsy of infancy with migrating focal seizures; EOEE = early-onset epileptic encephalopathy; NFLE = nocturnal frontal lobe epilepsy.

KCNT1 tetramers form a transmembrane sodium-activated potassium channel. Each subunit consists of 6 transmembrane domains with an extended cytoplasmic carboxy (C-) terminus (figure 4). The majority of reported pathogenic variants (table e-7, http://links.lww.com/WNL/A6), as seen in this study, are located in the C-terminus with clustering around the RCK and nicotinamide adenine dinucleotide–binding domains (figure 4). More recently, several variants have been identified within transmembrane domain 5 and in the pore-forming regions between transmembrane domains 4 and 5^4,5,8–10^ (table e-7), and this study also demonstrates epilepsy-associated mutations in transmembrane domains.

To date, different model systems have been used to determine the functional effects of *KCNT1* variants.^1,5,7,10,13,19,27^ Our protein homology structural modeling data predict abnormal gating or protein instability within the pore-forming region as a putative disease mechanism. In silico modeling of G288S has predicted similar detrimental effects,^5^ while Y775H is predicted to affect sodium sensitivity of the channel.^27^ *KCNT1* variants may therefore alter structural properties of the protein, contributing to altered channel function. Consistent with previous reports^1,3,13,19,27^ (table e-7, http://links.lww.com/WNL/A6), our *xenopus* oocyte model demonstrated that *KCNT1* pathogenic variants display a gain-of-function effect with increased current amplitude (figure 2). Previous studies have sought to correlate disease severity with the degree of gain of function.^1,19^ However, in keeping with more recent studies,^8^ such correlation was not evident in our study. *KCNT1* variants result in an increased P_o_ (probability of the channel being open), which may be due to increased mutant channel cooperativity or altered sodium sensitivity.^8,27^ In a recent study, sodium removal from the pipette solution had a less negative effect on G288S channel amplitude than WT, suggesting reduced sodium sensitivity in the mutant.^13^ Heterotetramer formation may be of importance in vivo. In 1 study, mutant KCNT1 homomers revealed a more marked gain of function than mutant WT heteromers.^13^ A significant remaining question is how *KCNT1* gain-of-function variants with predicted effects on neuronal hyperpolarization result in epilepsy.^28^ Altered voltage sensitivity may result in KCNT1 channels opening at more depolarized potentials, allowing a persistent hyperpolarizing current, with resultant interneuronal disinhibition as reported in *SCN1A*-related epilepsy.^39^ Conversely, increased repolarization permitting more frequent and rapid action potentials may also play a role.^27,34^

Recently, quinidine has been identified as a novel therapy for patients with *KCNT1*-related epilepsy. In in vitro models, quinidine has been shown to reduce the abnormal increase in mutant KCNT1 channel amplitude.^19^ For 1 patient with EIMFS with the *KCNT1* variant R428Q, in vitro testing showed quinidine sensitivity, and treatment resulted in a dramatic improvement in seizure control with neurodevelopmental gains.^3,7^ However, in more recent studies, patient response has been variable and not always as predicted by in vitro studies.^11^ Indeed, another patient with the same variant (R428Q) but different epilepsy phenotype (unclassified EOEE) failed to respond to quinidine, albeit at a later stage in the disease course.^14^ Most recently, a patient with West syndrome had a good response but only with a higher dose of 60 mg/kg/d.^2^ Clinical response may possibly be determined by the specific variant, other genetic factors, epilepsy phenotype, and drug timing within a therapeutic window. In our series, we treated 3 patients with quinidine, and 1 patient showed a clinical response. One patient developed a severe pulmonary vasculopathy, after which quinidine was discontinued. Systemic vasculitis has been reported with quinidine treatment.^40^ While investigations in our patient did not reveal overt evidence of vasculitis, the observed pulmonary dysfunction may represent an adverse drug-related event. The precise mechanism of KCNT1 blockade by quinidine is unclear, and it is possible that the disease mechanism for F346L, perhaps involving abnormal channel-opening dynamics as suggested by the modeling data, is not modifiable by quinidine. Our data suggest that quinidine should be considered as a therapeutic option for patients with *KCNT1* variants, but used with caution. Larger studies will provide further guidance about clinical utility, patient selection, optimum age at administration, and dose. Other KCNT1 modulators, including bepridil and clofililum, have been identified as possible alternative therapies.^28^ Like quinidine, bepridil has been shown in vitro to reversibly block mutant KCNT1 channels at a lower concentration than WT channels.^13^ However, similar to quinidine, potential cardiac effects and lack of specificity may limit use in patients.

Pathogenic variants in *KCNT1* cause a wide spectrum of severe epilepsies typically associated with impaired neurologic development and significant disease burden. As demonstrated, in vitro model systems may be useful to validate putative variants and to confirm pathogenicity, although genotype-phenotype correlations remain unclear. Evaluation of new therapies, including KCNT1-specific blockers, remains a research priority for this devastating pharmacoresistant group of epilepsies.

## Note added in proof

Recently, 3 patients with de novo *KCNT1* mutations and massive systemic to pulmonary collateral artery formation, presenting with pulmonary hemorrhage requiring embolization, were described. These patients had not been treated with quinidine. However, the mechanism remains unclear and further investigation of the expression and role of KCNT1 in the cardiovascular system is required.^41^

## Glossary

ADNFLE: autosomal dominant nocturnal frontal lobe epilepsy
EIMFS: epilepsy of infancy with migrating focal seizures
EOEE: early-onset epileptic encephalopathy
NFLE: nocturnal frontal lobe epilepsy
RCK: regulator of potassium conductance
SLACK: sequence like a calcium-dependent potassium channel
WT: wild-type
